# BIO FOr CARE: biomarkers of hypertrophic cardiomyopathy development and progression in carriers of Dutch founder truncating *MYBPC3* variants—design and status

**DOI:** 10.1007/s12471-021-01539-w

**Published:** 2021-02-02

**Authors:** M. Jansen, I. Christiaans, S. N. van der Crabben, M. Michels, R. Huurman, Y. M. Hoedemaekers, D. Dooijes, J. D. H. Jongbloed, L. G. Boven, R. H. Lekanne Deprez, A. A. M. Wilde, J. J. M. Jans, J. van der Velden, R. A. de Boer, J. P. van Tintelen, F. W. Asselbergs, A. F. Baas

**Affiliations:** 1 Department of Genetics, University Medical Centre Utrecht, Utrecht University, Utrecht, The Netherlands; 2 Department of Genetics, University Medical Centre Groningen, University of Groningen, Groningen, The Netherlands; 3 Department of Clinical Genetics, Amsterdam UMC, University of Amsterdam, Amsterdam, The Netherlands; 4 Department of Cardiology, Thoraxcenter, Erasmus University Medical Centre, Rotterdam, The Netherlands; 5 Department of Clinical Genetics, Radboud University Medical Centre, Nijmegen, The Netherlands; 6 Heart Centre, Clinical and Experimental Cardiology, Amsterdam UMC, University of Amsterdam, Amsterdam, The Netherlands; 7 Department of Physiology, Amsterdam UMC, Vrije Universiteit Amsterdam, Amsterdam Cardiovascular Sciences, Amsterdam, The Netherlands; 8 Department of Cardiology, University Medical Centre Groningen, University of Groningen, Groningen, The Netherlands; 9 Netherlands Heart Institute, Utrecht, The Netherlands; 10 Department of Cardiology, University Medical Centre Utrecht, Utrecht, The Netherlands; 11 Institute of Cardiovascular Science, Faculty of Population Health Sciences, University College London, London, UK; 12 Health Data Research UK and Institute of Health Informatics, University College London, London, UK

**Keywords:** Hypertrophic cardiomyopathy, MYBPC3, Biomarkers, Prognosis

## Abstract

**Background:**

Hypertrophic cardiomyopathy (HCM) is the most prevalent monogenic heart disease, commonly caused by truncating variants in the *MYBPC3* gene. HCM is an important cause of sudden cardiac death; however, overall prognosis is good and penetrance in genotype-positive individuals is incomplete. The underlying mechanisms are poorly understood and risk stratification remains limited.

**Aim:**

To create a nationwide cohort of carriers of truncating *MYBPC3* variants for identification of predictive biomarkers for HCM development and progression.

**Methods:**

In the multicentre, observational BIO FOr CARe (Identification of BIOmarkers of hypertrophic cardiomyopathy development and progression in Dutch *MYBPC3* FOunder variant CARriers) cohort, carriers of the c.2373dupG, c.2827C > T, c.2864_2865delCT and c.3776delA *MYBPC3* variants are included and prospectively undergo longitudinal blood collection. Clinical data are collected from first presentation onwards. The primary outcome constitutes a composite endpoint of HCM progression (maximum wall thickness ≥ 20 mm, septal reduction therapy, heart failure occurrence, sustained ventricular arrhythmia and sudden cardiac death).

**Results:**

So far, 250 subjects (median age 54.9 years (interquartile range 43.3, 66.6), 54.8% male) have been included. HCM was diagnosed in 169 subjects and dilated cardiomyopathy in 4. The primary outcome was met in 115 subjects. Blood samples were collected from 131 subjects.

**Conclusion:**

BIO FOr CARe is a genetically homogeneous, phenotypically heterogeneous cohort incorporating a clinical data registry and longitudinal blood collection. This provides a unique opportunity to study biomarkers for HCM development and prognosis. The established infrastructure can be extended to study other genetic variants. Other centres are invited to join our consortium.

**Supplementary Information:**

The online version of this article (10.1007/s12471-021-01539-w) contains supplementary material, which is available to authorized users.

## Introduction

Hypertrophic cardiomyopathy (HCM) is characterised by hypertrophy of the ventricular wall not explained by abnormal loading conditions [1]. HCM is an important cause of sudden cardiac death (SCD) [2] and may also lead to end-stage heart failure and left ventricular outflow tract (LVOT) obstruction [3]. The prevalence of HCM has historically been estimated at 1:500 [4], making it the most common Mendelian heart disease, with more recent estimates as high as 1:200 [5]. HCM is typically inherited as an autosomal dominant disease and a likely pathogenic/pathogenic variant is found in approximately 50% of patients [6, 7]. The most commonly affected gene is *MYBPC3*, which encodes cardiac myosin-binding protein C (cMyBP-C), an important regulator of cardiomyocyte contraction [3].

Despite the association with life-threatening arrhythmia and end-stage heart failure, clinical severity in HCM is highly variable, with low overall mortality [8] and incomplete, age-dependent penetrance in carriers of pathogenic variants (G+) [9]. This highlights the need for risk stratification. Current guidelines advocate the use of the HCM Risk-SCD calculator to identify patients who may benefit from a prophylactic implantable cardioverter-defibrillator [1, 10]. However, prediction of SCD remains imperfect [11] and risk-prediction models of other aspects of HCM have not yet been established. Furthermore, underlying mechanisms contributing to disease progression, such as environmental and additional (epi)genetic factors, are still unclear.

Similarly, prediction of penetrance in G+ phenotype-negative (LVH-) individuals is limited. Risk factors and biomarkers found in exploratory studies, including electrocardiographic abnormalities and impaired diastolic function on echocardiography [12–14], have not yet been validated in large, prospective cohorts. Without such data, these individuals undergo frequent cardiological screenings with a major impact on the health care system and costs.

The Dutch HCM population is relatively genetically homogeneous, with three *MYBPC3* founder variants (c.2373dupG (p.Trp792fs), c.2827C > T (p.Arg943Ter) and c.2864_2865delCT (p.Pro955fs), each inherited from a distant common ancestor) accounting for up to 35% of Dutch HCM cases [15]. Another truncating *MYBPC3* variant, c.3776delA (p.Gln1259fs), has been identified in multiple independently presenting HCM patients. These genetic variants result in truncated mRNA, absence of truncated cMyBP‑C protein and a reduction of functional cMyBP‑C (haploinsufficiency) [16], which impairs cardiomyocyte function [17], and have been shown to have a similar prognostic impact [18]. Together, they provide a unique opportunity to study effect modifiers free from confounding resulting from distinct genotypes.

In the BIO FOr CARe (Identification of BIOmarkers of hypertrophic cardiomyopathy development and progression in Dutch *MYBPC3* FOunder variant CARriers) study we aim to investigate predictive factors and effect modifiers for penetrance and progression of HCM in the genetically homogeneous group of Dutch carriers of truncating *MYBPC3* variants. Here we report the design and current status of this cohort and discuss ongoing and future studies.

## Methods

### Subject inclusion

**Fig. 1:**
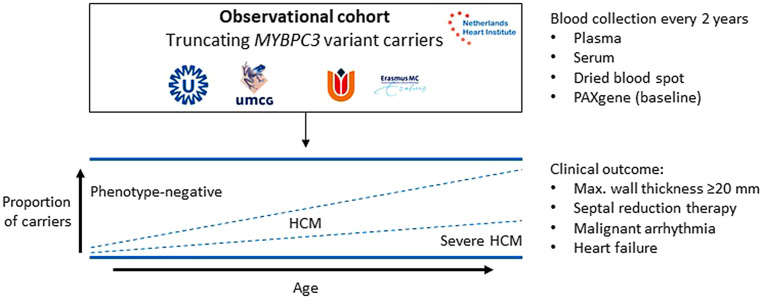
Schematic overview of the study design. The BIO FOr CARe (Identification of BIOmarkers of hypertrophic cardiomyopathy development and progression in Dutch *MYBPC3* FOunder variant CARriers) study comprises an observational cohort of Dutch carriers of the c.2373dupG (p.Trp792fs), c.2827C > T (p.Arg943Ter), c.2864_2865delCT (p.Pro955fs) and c.3776delA (p.Gln1259fs) variants in the *MYBPC3* gene. These variants are predicted to result in truncated mRNA with absence of truncated protein leading to haploinsufficiency. Subjects across the phenotypic spectrum associated with these variants are prospectively included for blood collection every 2 years and followed up over time. Clinical data are retrospectively collected from the first presentation onwards in a clinical registry to assess potential clinical predictors of disease penetrance and progression. *HCM* hypertrophic cardiomyopathy

Subjects are included as part of the ongoing prospective, multicentre, longitudinal, observational BIO FOr CARe cohort, embedded in the CVON DOSIS (Cardiovascular Research Netherlands—Determinants Of Susceptibility In inherited cardiomyopathy: towards novel therapeutic approacheS) consortium [19]. The design of this study is illustrated in Fig. 1. In short, clinical data from each subject’s first cardiomyopathy-related presentation onwards (due to symptoms or coincidental findings prompting echocardiography and/or cardiac magnetic resonance imaging (MRI), or screening for familial disease) are retrospectively collected in a registry and subjects prospectively undergo blood collection.

Subject inclusion started at the University Medical Centre Utrecht in January 2017, at the University Medical Centre Groningen in December 2017, at the Amsterdam University Medical Centre in January 2019 and is expected to commence shortly at the Erasmus Medical Centre in Rotterdam. G+ index patients (first family member to undergo genetic testing) and family members are identified through the genome diagnostics laboratories of each of these centres and screened for eligibility. Inclusion criteria are carriership of c.2373dupG, c.2827C > T, c.2864_2865delCT or c.3776delA variant in *MYBPC3* (reference sequence NM_000256.3) and age ≥ 18 years. Patients with prior heart transplantation are included in the clinical data registry, but are excluded from blood collection.

This study is performed in accordance with the Helsinki declaration and was approved by the Medical Ethics Committee of the UMC Utrecht. Informed consent is obtained from all subjects.

### Study outcome

The primary outcome of this study is a composite endpoint representing disease progression, consisting of (1) maximum wall thickness (MWT) ≥ 20 mm, (2) occurrence of heart failure (congestive heart failure or left ventricular ejection fraction < 50%), (3) LVOT obstruction (LVOT gradient ≥ 30 mm Hg with symptoms or ≥ 50 mm Hg regardless of symptoms) or (4) malignant arrhythmia (sustained ventricular tachycardia, ventricular fibrillation, appropriate implantable cardioverter-defibrillator intervention, cardiac arrest or SCD).

HCM is diagnosed according to the European Society of Cardiology criteria, summarised as a MWT of ≥ 15 mm in probands or ≥ 13 mm in first-degree family members, not proportional to abnormal loading conditions [1]. Dilated cardiomyopathy (DCM), which may likewise occur in carriers of *MYBPC3* variants, is diagnosed in accordance with the revised definition of the European Society of Cardiology myocardial and pericardial diseases working group [20]. Subjects fulfilling DCM criteria are carefully evaluated for signs of prior HCM (prior or persisting hypertrophy fulfilling diagnostic criteria for HCM).

### Study data

Study data are collected from electronic health records using a REDCap (Research Electronic Data Capture, Vanderbilt University, Nashville, TN, USA) tool hosted by the Netherlands Heart Institute. Baseline data include patient demographics, genetic analyses, family history, medical history, laboratory results, and conventional electrocardiography, ambulatory Holter monitoring, exercise test, echocardiography and cardiac MRI parameters. A comprehensive list of clinical variables is provided in the Electronic Supplementary Material (Table S1). The full data dictionary is available upon request. Clinical management and follow-up intervals remain at the discretion of the subject’s cardiologist.

### Statistical analysis and sample size calculation

All analyses were conducted in R version 4.0.0 (R Development Core Team, 2017) using RStudio version 1.1.414 (RStudio Team, 2015). Dichotomous variables are presented as counts with percentages and were analysed using two-sided Fisher’s exact test. Continuous variables are presented as means ± standard deviations or medians with interquartile ranges according to their distribution and analysed using unpaired *t*- or Mann-Whitney U tests accordingly.

According to the ‘one in ten’ rule of thumb for regression analyses, 30 subjects would have to fulfil the primary outcome to assess one biomarker and correct for two variables (age and sex). Assuming 40% of enrolled subjects to be G + LVH- at baseline, 75% of these G + LVH- subjects to develop a phenotype throughout life and 10% of those developing a phenotype to fulfil the primary outcome, a total of 1000 subjects are required to enrol in the study.

### Blood collection

Standardised blood collection is performed to study potential biomarkers for HCM development and progression. Using 23G winged blood collection sets, venous blood samples are collected and processed within 45 min to obtain 4 × 500 µl plasma from citrate tubes, 6 × 500 µl plasma and a dried (whole) blood spot card from lithium-heparin tubes, 6 × 500 µl serum and a whole-blood PAXgene RNA tube (PreAnalytiX GmbH, Hombrechtikon, Switzerland). The laboratory protocol is provided in the Electronic Supplementary Material (Fig. S1).

## Preliminary results

**Fig. 2:**
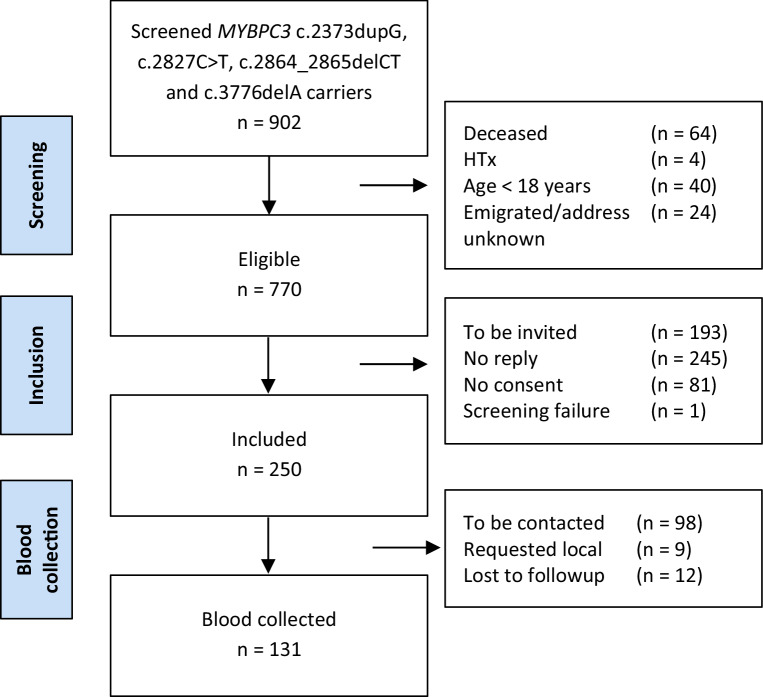
Flowchart showing the results of subject screening, as well as data on inclusion and blood collection. The reasons for exclusion are noted on the right for each stage. *Requested local* denotes subjects who requested blood collection in local cardiological centres. *HTx* heart transplantation

A flowchart outlining subject inclusion and follow-up is presented in Fig. 2. A total of 250 G+ individuals were included in the study up to 1 January 2020. In 232 subjects the diagnosis could be ascertained. HCM was diagnosed in 169 subjects and DCM without prior diagnosis of HCM in 4 subjects. The remaining 59 subjects were G + LVH- at the time of inclusion. Baseline characteristics are presented in Tab. 1. A male predominance (66%) was observed among index patients, similar to previous studies [21, 22]. To date, blood samples have been obtained from 131 subjects. As shown in the Electronic Supplementary Material (Table S2), the baseline characteristics of subjects who have undergone blood collection and those who have not are similar.

**Table 1:** Subject characteristics

		Overall	Index patient	Family member	*p*-value
		(*n* = 250)	(*n* = 97)	(*n* = 153)	
*Demographics*					
Age at inclusion (years)		54.9 [43.3, 66.6]	55.5 [47.3, 67.2]	53.7 [40.5, 64.5]	0.183
Male sex		137 (54.8)	64 (66.0)	73 (47.7)	** 0.007**
Body surface area (m^2^)		1.96 [1.83, 2.13]	1.95 [1.85, 2.19]	1.96 [1.82, 2.10]	0.448
*Genetics*					
*MYBPC3* variant	c.2373dupG	185 (74.0)	69 (71.1)	116 (75.8)	0.500
	c.2827C > T	30 (12.0)	14 (14.4)	16 (10.5)	0.425
	c.2864_2865delCT	15 (6.0)	5 (5.2)	10 (6.5)	0.788
	c.3776delA	20 (8.0)	9 (9.3)	11 (7.2)	0.634
*Patient history*					
Syncope		28 (11.8)	15 (16.1)	13 (9.0)	0.103
NYHA class	I/II	144 (93.5)	52 (88.1)	92 (96.8)	** 0.045**
	III/IV	10 (6.5)	7 (11.9)	3 (3.2)	
Family history of sudden cardiac death	Any first-degree family member	86 (36.0)			0.489
	In accordance with ESC HCM Risk-SCD calculator	45 (22.1)			0.300
*Holter monitoring*					
Non-sustained ventricular tachycardia		102 (57.0)	51 (65.4)	51 (50.5)	0.050
*Imaging*					
Maximum wall thickness (mm)		16 [12, 20]	19 [17, 23]	13 [10, 17]	**<0.001**
Maximum LV outflow tract gradient (mm Hg)		6 [4, 10]	8 [5, 15]	5 [4, 7]	** 0.003**
LV end-diastolic diameter (mm)		46 [40, 50]	45 [40, 49]	46 [41, 50]	0.438
LV ejection fraction (%)		60 [55, 62]	60 [52, 62]	60 [55, 63]	0.232
LV diastolic dysfunction	Normal	69 (59.5)	13 (36.1)	56 (70.0)	**<0.001**
	Impaired relaxation	29 (25.0)	10 (27.8)	19 (23.8)	
	Pseudonormalisation	11 (9.5)	9 (25.0)	2 (2.5)	
	Restrictive	7 (6.0)	4 (11.1)	3 (3.8)	
LA diameter (mm)		41 [36, 46]	42 [37, 46]	39 [36, 46]	0.533
*Outcomes at inclusion*					
Phenotype	Phenotype-negative	59 (25.4)	3 (3.2)^a^	56 (40.6)	**<0.001**
	HCM 13–14 mm	18 (7.8)	1 (1.1)	17 (12.3)	
	HCM ≥ 15 mm	151 (65.1)	88 (93.6)	63 (45.7)	
	DCM	4 (1.7)	2 (2.1)	2 (1.4)	
Primary outcome	Composite endpoint	115 (50.2)	71 (78.0)	44 (31.7)	**<0.001**
	Maximum wall thickness ≥ 20 mm	88 (39.8)	58 (65.9)	30 (22.4)	**<0.001**
	Septal reduction therapy	17 (7.8)	14 (16.1)	3 (2.3)	**<0.001**
	Malignant ventricular arrhythmia	21 (9.3)	14 (15.4)	7 (5.1)	** 0.011**
	Heart failure	47 (22.0)	30 (34.9)	17 (13.2)	**<0.001**
	Congestive heart failure	28 (13.6)	17 (21.8)	11 (8.5)	** 0.011**
	Systolic heart failure	33 (16.0)	20 (26.3)	13 (9.9)	** 0.003**

*NYHA* New York Heart Association, *ESC* European Society of Cardiology, *SCD* sudden cardiac death, *LV* left ventricular, *LA* left atrial, *HCM* hypertrophic cardiomyopathy, *DCM* dilated cardiomyopathy

Continuous data are shown as means ± standard deviation for normally distributed variables or median [interquartile range] for non-normally distributed variables. Dichotomous and categorical data are shown as counts (% of valid). *p*-values < 0.05 are depicted in bold

^a^Incidental finding in one subject and genetic testing prompted by a sudden cardiac death of a family member in two subjects

## Ongoing and future projects

Currently, two biomarker discovery studies are ongoing in the BIO FOr CARe cohort, both utilising metabolomics approaches. Previous studies have indicated a perturbed energy metabolism in HCM with an increased adenosine triphosphate utilisation and decreased energetic efficiency [16, 23]. Acylcarnitines are forms of fatty acids transported into mitochondria for energy production [24]. Changes in acylcarnitine concentrations have been shown to reflect a shift away from the fatty acid oxidation that is normally predominant in cardiac tissue, towards glucose utilisation in a *MYBPC3* HCM mouse model treated with perhexiline (which blocks transportation of fatty acids into mitochondria) [25]. This shift to increased glucose utilisation is more generally observed in heart failure [26], in which increased levels of long-chain acylcarnitines have been associated with worse prognosis [27].

In the BIO FOr CARe study, acylcarnitines were determined using targeted metabolomics in 121 plasma samples, together with 27 samples collected prior to genetic testing as part of the *UNRAVEL* biobank [28], including 12 genotype-negative relatives not eligible for inclusion in BIO FOr CARe. To further study changes in the metabolome associated with HCM, untargeted metabolomics using a previously published direct-infusion high-resolution mass spectrometry platform [29] was performed in a nested case-control study, consisting of 30 subjects with a severe phenotype and 30 age- and sex-matched subjects with a mild or no phenotype, as well as 10 age- and sex-matched genotype-negative family members from the UNRAVEL biobank.

Additionally, an exploratory proteomics study using the Olink Cardiovascular III multiplex immunoassay (for more information, visit: https://www.olink.com/products/cvd-iii-panel/) is being designed. Furthermore, the BIO FOr CARe infrastructure is currently being used for observational cohort studies involving other HCM genotypes and for gene-expression studies in induced pluripotent stem-cell-derived cardiomyocytes, and will be used for potential future genome-wide association studies/polygenic risk score analyses.

## Discussion

The BIO FOr CARe cohort offers a unique opportunity to investigate predictive factors and effect modifiers for penetrance and progression of HCM. Currently, 250 carriers of truncating *MYBPC3* variants across the phenotypic spectrum have been included. Blood samples have been obtained from 131 subjects and several biomarker discovery studies are currently underway.

Standardisation of blood collection allows for future biomarker studies requiring plasma, serum or RNA. By repeating blood collection at subsequent time points, changes in biomarker concentrations can be validated over time and across progressive stages of the phenotype. The current biomarker studies are still limited to a cross-sectional design. However, longitudinal assessment of biomarkers will become possible over time, as disease onset and progression may occur in carriers during follow-up.

The prognostic utility of biomarkers in HCM is also being studied in several other studies, such as the HCMR—Novel Markers of Prognosis in Hypertrophic Cardiomyopathy (HCMR) study (ClinicalTrials.gov Identifier: NCT01915615) [30], the Predictive Factors and Consequences of Myocardial Fibrosis in Hypertrophic Cardiomyopathy (HCM) study (NCT02922517), the Cardiac Biomarkers in Pediatric Cardiomyopathy (PCM Biomarkers) study (NCT01873976), and the An Integrative-‘Omics’ Study of Cardiomyopathy Patients for Diagnosis and Prognosis in China (AOCC) study (NCT03076580). Our study distinguishes itself by its genotype-driven study population, including carriers of pathogenic variants regardless of their phenotype, and its longitudinal design. The inclusion of G + LVH- individuals is made possible by the widespread availability of genetic screening to family members (covered by the national basic healthcare plan), which has led to the identification of many G + LVH-family members [7]. This provides a unique opportunity to study biomarkers and their prognostic utility at subsequent stages of disease progression. Furthermore, the high prevalence of *MYBPC3* founder variants in the Dutch HCM population allows analyses free from confounding resulting from genetic heterogeneity [15].

Additionally, the ancillary REDCap registry offers a user-friendly, sustainable platform for clinical data collection using standardised definitions. Similar to the Netherlands Arrhythmogenic Cardiomyopathy Registry [31], this may serve as a foundation for future observational studies, involving carriers of other variants and other genetic cardiomyopathies. A wide range of data is collected from the subject’s first presentation onwards, enabling both cross-sectional and longitudinal studies as well as hypothesis-driven and hypothesis-generating approaches. New variables can easily be added to accommodate studies focussing on specific disease aspects.

Both data and samples are available to external researchers through submission of an application to our data access board, which consists of investigators from each participating centre. So far, inclusions have focussed on the genetically homogeneous cohort of pathogenic truncating *MYBPC3* variants. Although the prognosis of HCM patients carrying founder variants in *MYBPC3* has been shown to be similar to other G + HCM [18, 21], findings in our current cohort will have to be validated in the general HCM population. An amendment to the BIO FOr CARe protocol to include all HCM genotypes has recently been accepted. External validation may be performed in large multi-national cohorts such as the HCMR study [30]. Potenzial effect modifiers may be further studied in myocardial samples collected from septal myectomies or during heart transplantation.

A limitation of the current study design is the risk of referral bias, as blood collection is limited to academic hospitals, which may hinder patients who are under cardiological follow-up elsewhere and do not live nearby. This likely contributed to the higher than anticipated number of subjects fulfilling the primary outcome at baseline (50%). Extending blood collection to regional hospitals would ameliorate the potential bias. A second limitation is that clinical data were not collected prospectively, resulting in missing data. A further limitation to this study is that event rates are low in HCM [8]. As a result, a large sample size and long follow-up duration are required for prospective and longitudinal studies, for which embedding in a sustainable infrastructure and long-term funding are essential. Finally, as the subjects in this study were ascertained as index patients or through their relatedness to one, results may not be generalisable to carriers of *MYBPC3* variants in the general population. Instead, population-based studies are required to investigate the prognosis of carriers in the general population.

## Conclusion

The BIO FOr CARe cohort incorporates a clinical data registry and standardised prospective blood sample collection, offering a unique opportunity to investigate factors affecting development and progression of HCM in carriers of truncating founder *MYBPC3* variants. The cohort comprises patients across the phenotypic spectrum, allowing cross-sectional biomarker studies, of which several are under way. Longitudinal collection of blood samples and clinical data allows prospective and longitudinal assessment of biomarkers at progressive stages of HCM. The established collaborations and infrastructure can be extended to study other genotypes and different genetic cardiomyopathies. Other centres are invited to join our consortium.

## Supplementary Information

Registry variables, baseline characteristics stratified by blood collection, lab protocol.
