# Supplementary material for: BIO FOr CARE: biomarkers of hypertrophic cardiomyopathy development and progression in carriers of Dutch founder truncating MYBPC3 variants—design and status
Source: Neth Heart J. 2021 Feb 2;29(6):318–29. doi: 10.1007/s12471-021-01539-w (PMC8160056; doi:10.1007/s12471-021-01539-w)
Supplement: Supplementary file 1 — Registry variables, baseline characteristics stratified by blood collection, lab protocol. [file 12471_2021_1539_MOESM1_ESM.docx]

**Table S1: Registry variables**

| **Form Name** | **Field Label** | **Choices** |
| --- | --- | --- |
| Demographics | Included studies | UMC Utrecht UNRAVEL registry/biobank \| BIO FOr CARe prospective cohort \| Exercise interview |
|  | Inclusion date(s) (per study) |  |
|  | Inclusion centre | UMCU \| UMCG \| AMC \| Erasmus MC |
|  | Year of Birth |  |
|  | Sex | Male \| Female \| Unknown |
|  | Place family tree | Proband \| Family member \| Unknown |
|  | Relationship to proband |  |
|  | Overall family ID |  |
| Genetics | Date of (first) genetic analysis |  |
|  | Genetics Lab | AMC \| EMC \| LUMC \| MUMC \| UMCG \| UMCU \| RUMC \| Other \| Unknown |
|  | Method of DNA testing | Panel \| Sequential \| Variant-specific \| Unknown |
|  | Number of Genes tested |  |
|  | Mutated gene | MYBPC3 \| MYH7 \| TNNT2 \| TNNI3 \| TPM1 \| ACTC1 \| MYL2 \| MYL3 \| Other |
|  | Reference sequence (per gene) |  |
|  | Gene variant (c.) (per gene) |  |
|  | Gene variant (p.) (per gene) |  |
|  | Variant classification (class) (per gene) | Pathogenic (class 5) \| likely pathogenic (class 4) \| unclassified variant (class 3) |
| Family history | Date at which family history was ascertained |  |
|  | Affected first-degree relative? | Yes \| No \| Unknown |
|  | HCM in first-degree relative? | Yes \| No \| Unknown |
|  | Youngest age HCM first-degree |  |
|  | DCM in first-degree relative? | Yes \| No \| Unknown |
|  | Youngest age DCM first-degree |  |
|  | NCCM in first-degree relative? | Yes \| No \| Unknown |
|  | Youngest age NCCM first-degree |  |
|  | Septal reduction therapy in first-degree relative? | Yes \| No \| Unknown |
|  | Youngest age septal reduction therapy first-degree |  |
|  | Heart failure in first-degree relative? | Yes \| No \| Unknown |
|  | Youngest age heart failure first-degree |  |
|  | Sudden cardiac death in first-degree relative? | Yes \| No \| Unknown |
|  | Youngest age sudden cardiac death first-degree |  |
|  | Sudden cardiac death family member known with cardiomyopathy? | Yes \| No \| Unknown |
|  | Affected second-degree relative? | Yes \| No \| Unknown |
|  | HCM in second-degree relative? | Yes \| No \| Unknown |
|  | Youngest age HCM second-degree |  |
|  | DCM in second-degree relative? | Yes \| No \| Unknown |
|  | Youngest age DCM second-degree |  |
|  | NCCM in second-degree relative? | Yes \| No \| Unknown |
|  | Youngest age NCCM second-degree |  |
|  | Septal reduction therapy in second-degree relative? | Yes \| No \| Unknown |
|  | Youngest age septal reduction therapy second-degree |  |
|  | Heart failure in second-degree relative? | Yes \| No \| Unknown |
|  | Youngest age heart failure second-degree |  |
|  | Sudden cardiac death in second-degree relative? | Yes \| No \| Unknown |
|  | Youngest age sudden cardiac death second-degree |  |
|  | Affected family >2nd degree? | Yes \| No \| Unknown |
|  | HCM in family >2nd degree? | Yes \| No \| Unknown |
|  | Age youngest HCM >2nd degree |  |
|  | DCM in family >2nd degree? | Yes \| No \| Unknown |
|  | Age youngest DCM >2nd degree |  |
|  | NCCM in family >2nd degree? | Yes \| No \| Unknown |
|  | Age youngest NCCM >2nd degree |  |
|  | Septal reduction therapy in family >2nd degree? | Yes \| No \| Unknown |
|  | Age youngest septal reduction therapy >2nd degree |  |
|  | Heart failure in family >2nd degree? | Yes \| No \| Unknown |
|  | Age youngest heart failure >2nd degree |  |
|  | Sudden cardiac death in family >2nd degree? | Yes \| No \| Unknown |
|  | Age youngest SCD >2nd degree |  |
| Comorbidities & intoxications | Date comorbidities reported |  |
|  | Hypertension | Yes \| No \| Unknown |
|  | Date of diagnosis hypertension |  |
|  | Diabetes mellitus | Yes, type I \| Yes, type II \| Yes, not otherwise defined \| No \| Unknown |
|  | Date of diagnosis diabetes mellitus |  |
|  | Adipositas | Yes \| No \| Unknown |
|  | Date of diagnosis adipositas |  |
|  | Dyslipidaemia | Yes \| No \| Unknown |
|  | Date of diagnosis dyslipidaemia |  |
|  | Myocardial infarction | Yes \| No \| Unknown |
|  | Date of diagnosis myocardial infarction |  |
|  | Stroke | transient ischemic attack \| cerebrovascular accident \| Yes, not otherwise defined \| No \| Unknown |
|  | Date of diagnosis transient ischemic attack |  |
|  | Date of diagnosis cerebrovascular accident |  |
|  | Date of diagnosis stroke not otherwise defined |  |
|  | Peripheral vascular disease | Yes \| No \| Unknown |
|  | Date of diagnosis peripheral vascular disease |  |
|  | Cardiac sarcoidosis | Yes \| No \| Unknown |
|  | Date of diagnosis cardiac sarcoidosis |  |
|  | Cardiac amyloidosis | Yes \| No \| Unknown |
|  | Date of diagnosis cardiac amyloidosis |  |
|  | Other comorbidities | Yes \| No \| Unknown |
|  | Chronic obstructive pulmonary disease | Yes, GOLD I \| Yes, GOLD II \| Yes, GOLD III \| Yes, GOLD IV \| Yes, not otherwise defined \| No \| Unknown |
|  | Date of diagnosis chronic obstructive pulmonary disease |  |
|  | Obstructive sleep apnoea syndrome | Yes \| No \| Unknown |
|  | Date of diagnosis obstructive sleep apnoea syndrome |  |
|  | Other diagnoses |  |
|  | Date intoxications reported |  |
|  | Smoking | Yes \| No \| Unknown |
|  | Year started smoking |  |
|  | Year stopped smoking |  |
|  | Cigarettes per week |  |
|  | Packyears |  |
|  | Alcohol | Yes \| No \| Unknown |
|  | Alcohol units per week |  |
|  | Drugs | Yes, cocaine \| Yes, cannabis \| Yes, other \| No \| Unknown |
|  | Cocaine per week |  |
|  | Cannabis per week |  |
|  | Specify other drug |  |
|  | Other drugs per week |  |
|  | Sport enhancing substances | Yes, anabolic steroids \| Yes, testosterone \| Yes, creatine \| Yes, protein supplements \| Yes, other \| No \| Unknown |
|  | Anabolic steroids per week |  |
|  | Testosterone per week |  |
|  | Creatine per week |  |
|  | Protein supplement per week |  |
|  | Specify other |  |
|  | Other sport enhancing substance per week |  |
| Presentation | Centre of presentation |  |
|  | Date of presentation |  |
|  | Type of Presentation | Sudden cardiac death \| Symptomatic and living-not resuscitated (including those presenting with sustained VT and cardiac syncope) \| resuscitated cardiac arrest (witness collapse and revival with CPR) \| Abnormal test \| Family history \| Scheduled follow-up |
|  | Specify abnormal test | Auscultation \| ECG \| exercise ECG \| Holter \| Echo \| MRI |
|  | Ventricular tachycardia/fibrillation at presentation | Ventricular tachycardia \| Ventricular Fibrillation \| None \| Unknown |
|  | Symptoms at presentation | Syncope \| Presyncope \| Palpitations \| Chest pain \| Fatigue \| Dyspnoea (d'effort) \| Orthopnoea \| Oedema \| Weight gain \| Other |
|  | NYHA class at presentation | NYHA I \| NYHA II \| NYHA III \| NYHA IV \| Unknown |
|  | Betablocker | Yes \| No \| Unknown |
|  | Specify betablocker and dosage |  |
|  | Calcium antagonist (non-dihydropyridine) | Yes \| No \| Unknown |
|  | Specify calcium antagonist and dosage |  |
|  | Other antiarrhythmic | Yes \| No \| Unknown |
|  | Specify antiarrhythmic and dosage |  |
|  | Diuretic (including MRA) | Yes \| No \| Unknown |
|  | Specify diuretic and dosage |  |
|  | Ace-inhibitor/angiotensin receptor blocker | Yes \| No \| Unknown |
|  | Specify ace-inhibitor/angiotensin receptor blocker and dosage |  |
|  | Nitrate | Yes \| No \| Unknown |
|  | Specify nitrate and dosage |  |
|  | Other relevant drugs | Yes \| No \| Unknown |
|  | Specify other relevant drugs and dosage |  |
|  | Height |  |
|  | Weight |  |
|  | Systolic blood pressure |  |
|  | Diastolic blood pressure |  |
| ECG | Date ECG |  |
|  | Rhythm | Sinus rhythm \| Atrial pacing \| (atrial-)ventricular pacing \| Supraventricular tachycardia \| Other |
|  | Specify supraventricular tachycardia | Atrial fibrillation \| Atrial flutter \| Atrial tachycardia \| AV-nodal re-entry tachycardia \| AV re-entry tachycardia - orthodrome \| AV re-entry tachycardia - antidrome \| AV-junctional tachycardia |
|  | Heart Rate frequency |  |
|  | PQ interval |  |
|  | QRS duration |  |
|  | Axis |  |
|  | Conduction block | Typical cRBBB \| Atypical cRBBB \| iRBBB \| cLBBB \| iLBBB \| Aspecific intraventricular conduction delay \| AV-block 1 \| AV-block 2 Wenckebach \| AV-block 2 Mobitz \| AV-block 3 \| No \| Unknown |
|  | Leads pathological Q wave | V1 \| V2 \| V3 \| V4 \| V5 \| V6 \| I \| II \| III \| aVL \| aVR \| aVF \| Not specified |
|  | Leads T-wave inversion | V1 \| V2 \| V3 \| V4 \| V5 \| V6 \| II \| III \| aVF \| Not specified |
| Holter monitoring | Date Holter |  |
|  | Monitoring time |  |
|  | Total premature ventricular contraction amount |  |
|  | Non-sustained ventricular tachycardia | Yes \| No \| Unknown |
|  | Beats longest |  |
|  | Frequency fastest |  |
|  | Sustained ventricular tachycardia | Yes \| No \| Unknown |
| Echocardiography | Date echocardiography |  |
|  | Body surface area |  |
|  | LA dilatation | Yes \| No \| Unknown |
|  | Severity of LA dilatation | Mild \| Moderate \| Severe \| Unknown |
|  | LA diameter |  |
|  | LA volume |  |
|  | Indexed LA volume |  |
|  | Global LV dilatation | Yes \| No \| Unknown |
|  | Severity of LV dilatation | Mild \| Moderate \| Severe \| Unknown |
|  | Global LV dysfunction | Yes \| No \| Unknown |
|  | Severity of LV dysfunction | Mild \| Moderate \| Severe \| Unknown |
|  | Global LV diastolic dysfunction | Yes \| No \| Unknown |
|  | Severity of LV diastolic dysfunction | Normal \| Grade 1 impaired relaxation \| Grade 2 pseudonormal \| Grade 3/4 restrictive\| Unknown |
|  | LV (incl. septum) maximum wall thickness |  |
|  | Intraventricular septum thickness |  |
|  | Posterior wall thickness |  |
|  | Pattern of hypertrophy (reported) | Apical \| Asymmetric septal \| Concentric \| Mid/papillary muscle \| Sigmoidal \| Symmetric \| Unknown |
|  | LV end-diastolic diameter |  |
|  | LV end-systolic diameter |  |
|  | LV ejection fraction |  |
|  | LV ejection fraction method used | Eyeballing \| MOD-bp \| 3D \| Teich (preferably avoid) |
|  | Aortic valve stenosis | Normal \| Grade 1 mild \| Grade 2 moderate \| Grade 3 severe \| Prosthesis \| Unknown |
|  | Mitral valve regurgitation | Normal \| Grade 1 mild \| Grade 2 moderate \| Grade 3 severe \| Prosthesis \| Unknown |
|  | Mitral valve systolic anterior motion during rest | Yes \| No \| Unknown |
|  | Mitral valve systolic anterior motion during provocation | Yes \| No \| Unknown |
|  | Mitral E-wave |  |
|  | E-wave deceleration time |  |
|  | Mitral A-wave |  |
|  | Medial (septal) e' |  |
|  | Lateral e' |  |
|  | Average E/e' (reported) |  |
|  | Ao velocity max at rest |  |
|  | Ao gradient at rest |  |
|  | LV velocity max at rest |  |
|  | Left ventricular tract gradient at rest |  |
|  | Ao velocity max at provocation |  |
|  | Ao gradient at provocation |  |
|  | LV velocity max at provocation |  |
|  | Maximum left ventricular tract gradient at provocation |  |
| Cardiac MRI | Date MRI |  |
|  | Body surface area |  |
|  | LA diameter (x) |  |
|  | LA diameter (y) |  |
|  | LV end-diastolic diameter |  |
|  | LV end-diastolic volume |  |
|  | LV end-systolic diameter |  |
|  | LV end-systolic volume |  |
|  | LV ejection fraction |  |
|  | LV (incl. septum) maximum wall thickness |  |
|  | Intraventricular septum thickness |  |
|  | Anterior wall thickness |  |
|  | Lateral wall thickness |  |
|  | Inferior wall thickness |  |
|  | Posterior wall thickness |  |
|  | Pattern of hypertrophy | Apical \| Asymmetric septal \| Mid/papillary muscle \| Sigmoidal \| Symmetric \| Upper septal |
|  | RV end-diastolic volume |  |
|  | RV ejection fraction |  |
|  | Mitral valve regurgitation fraction |  |
|  | Late enhancement performed | Yes \| No \| Unknown |
|  | RV late enhancement present | Yes \| No \| Unknown |
|  | LV late enhancement present | Yes \| No \| Unknown |
|  | Extensive late enhancement, either quantified >=15% of LV mass or visually estimated as extensive/diffuse | Yes \| No \| Unknown |
|  | Late enhancement localization |  |
| Exercise test | Date exercise test |  |
|  | Absolute workload achieved |  |
|  | METS achieved |  |
|  | Peak heartrate achieved |  |
|  | Percentage of predicted max heartrate |  |
|  | Baseline systolic blood pressure |  |
|  | Baseline diastolic blood pressure |  |
|  | Systolic blood pressure at peak exercise |  |
|  | Diastolic blood pressure at peak exercise |  |
|  | Non-sustained ventricular tachycardia | Yes \| No \| Unknown |
| Laboratory markers | Date laboratory testing |  |
|  | Sodium |  |
|  | Potassium |  |
|  | Urea |  |
|  | Creatinine |  |
|  | Estimated glomerular filtration rate (eGFR) |  |
|  | Uric acid |  |
|  | Lactate |  |
|  | Alkaline phosphatase |  |
|  | Gamma-glutamyl transpeptidase (γGT) |  |
|  | Aspartate aminotransferase (ASAT) |  |
|  | Alanine aminotransferase (ALAT) |  |
|  | Lactate dehydrogenase (LD) |  |
|  | Creatine Kinase |  |
|  | Creatine Kinase myocardial band (CK-Mb) |  |
|  | Albumin |  |
|  | C-reactive protein (CRP) |  |
|  | Troponin T |  |
|  | High-sensitive Troponin T |  |
|  | Troponin I |  |
|  | High-sensitive Troponin I |  |
|  | Brain natriuretic peptide (BNP) |  |
|  | N-terminal prohormone of brain natriuretic peptide (NT-proBNP) |  |
|  | Total cholesterol |  |
|  | Triglycerides |  |
|  | High density lipoprotein cholesterol (HDL) |  |
|  | Low density lipoprotein cholesterol (LDL) |  |
|  | non-HDL cholesterol |  |
|  | Glucose |  |
|  | Glycated haemoglobin (HbA1c) |  |
|  | Haemoglobin |  |
|  | Haematocrit |  |
|  | Mean corpuscular volume (MCV) |  |
|  | Thrombocytes |  |
|  | Leukocytes |  |
|  | Date UNRAVEL sampling performed |  |
|  | Date BIO FOr CARe sampling |  |
| Diagnosis | Diagnosis | Unaffected \| Borderline HCM (maximum wall thickness 13-14mm) \| LV dilatation \| LVNC (noncompacted myocardium to compacted myocardium ratio on MRI >2.3 end-diastole, echo >2.0 end-systole) \| HCM (maximum wall thickness >=15mm, including those progressing to DCM) \| DCM (LV dilatation with LVEF< 45% or FS< 25%) \| NCCM (LVNC with LVEF< 50%, including those progressing to DCM) \| Ventricular tachycardia/ventricular fibrillation/sudden cardiac death not otherwise defined (no known diagnosis) \| Unknown |
|  | Date of diagnosis (per diagnosis) |  |
| Atrial arrhythmia | Did the patient experience an atrial arrhythmia | Yes \| No \| Unknown |
|  | Date of (first registration of) atrial arrhythmia |  |
| ICD implantation | Did the patient undergo ICD implantation | Yes \| No \| Unknown |
|  | Date of ICD implantation |  |
|  | Type of prevention | Primary \| Secondary \| Unknown |
| Outcome | Maximum wall thickness >= 20mm | Yes \| No \| Unknown |
|  | Date earliest |  |
|  | LV outflow tract obstruction >=50 mmHg or >=30 mmHg with symptoms necessitating treatment occurred | Yes \| No \| Unknown |
|  | Date earliest |  |
|  | Has myectomy been performed | Yes \| No \| Unknown |
|  | Date earliest |  |
|  | Has septal ablation been performed | Yes \| No \| Unknown |
|  | Date earliest |  |
|  | Has patient ever experienced heart failure | Yes \| No \| Unknown |
|  | Date earliest |  |
|  | Congestive heart failure | Yes \| No \| Unknown |
|  | Date earliest |  |
|  | Potential trigger for congestive heart failure |  |
|  | Has patient ever been hospitalised for heart failure | Yes \| No \| Unknown |
|  | Date earliest |  |
|  | Systolic heart failure (LV ejection fraction<50%) | Yes \| No \| Unknown |
|  | Date earliest |  |
|  | Has patient ever experienced stroke | Yes \| No \| Unknown |
|  | Date earliest |  |
|  | Has a pacemaker been implanted | Yes \| No \| Unknown |
|  | Date earliest |  |
|  | Has cardiac resynchronisation therapy device (pacemaker or defibrillator) been implanted | Yes \| No \| Unknown |
|  | Date earliest |  |
|  | Has LV assist device been implanted | Yes \| No \| Unknown |
|  | Date earliest |  |
|  | Has heart transplantation been performed | Yes \| No \| Unknown |
|  | Date earliest |  |
|  | Have ventricular tachycardia/fibrillation occurred | Yes \| No \| Unknown |
|  | Date earliest |  |
| Death | Is the patient deceased | Yes \| No \| Unknown |
|  | Date |  |
|  | Cardiac cause | Yes \| No \| uncertain |
|  | Specify cardiac cause | End-stage heart failure \| Ventricular arrhythmia (including sudden cardiac death) \| Other \| Unknown |
|  | Cause of death |  |
| Last follow-up/ last edited | Last follow-up |  |
|  | Date last edited |  |
|  | Remarks |  |

Comprehensive list of the clinical parameters collected through the electronic case report file of our REDCap registry. HCM, hypertrophic cardiomyopathy; DCM, dilated cardiomyopathy; NCCM, non-compaction cardiomyopathy; GOLD, global initiative for chronic obstructive lung disease; NYHA, New York heart association; cRBBB, complete right bundle branch block; iRBBB, incomplete right bundle branch block; cLBBB, complete left bundle branch block; iLBBB, incomplete left bundle branch block; LA, left atrial; LV, left ventricular; Ao, aortic; LVNC, left ventricular non-compaction; ICD, implantable cardioverter-defibrillator

**Table S2: Baseline characteristics stratified by blood collection**

|  |  | **Blood sample collected** | **Blood sample not yet collected** | **p-value** |
| --- | --- | --- | --- | --- |
|  |  | (n = 131) | (n = 119) |  |
| **Demographics** |  |  |  |  |
| Age at inclusion (years) |  | 53.7 [42.2, 67.3] | 56.5 [46.4, 64.0] | 0.391 |
| Male sex |  | 67 (51.1) | 70 (58.8) | 0.275 |
| Body surface area (m^2^) |  | 1.94 [1.81, 2.12] | 1.98 [1.85, 2.18] | 0.268 |
|  |  |  |  |  |
| **Genetics** |  |  |  |  |
| Index patient |  | 51 (38.9) | 44 (37.0) | 0.976 |
| *MYBPC3* pathogenic variant | c.2373dupG | 101 (77.1) | 84 (70.6) | 0.304 |
|  | c.2827C>T | 13 (9.9) | 17 (14.3) | 0.333 |
|  | c.2864_2865delCT | 12 (9.2) | 3 (2.5) | **0.033** |
|  | c.3776delA | 5 (3.8) | 15 (12.6) | **0.018** |
|  |  |  |  |  |
| **Patient history** |  |  |  |  |
| Syncope |  | 17 (13.2) | 11 (10.1) | 0.547 |
| NYHA class | I/II | 86 (92.5) | 58 (95.1) | 0.741 |
|  | III/IV | 7 (7.5) | 3 (4.9) |  |
| Family history of  sudden cardiac death | Any first-degree family member | 47 (36.4) | 39 (35.5) | 0.893 |
|  | In accordance with ESC HCM  Risk-SCD | 28 (24.6) | 17 (18.9) | 0.396 |
|  |  |  |  |  |
| **Holter monitoring** |  |  |  |  |
| Non-sustained ventricular tachycardia |  | 59 (59.6) | 43 (53.8) | 0.451 |
|  |  |  |  |  |
| **Imaging** |  |  |  |  |
| Maximum wall thickness (mm) |  | 15 [11, 19] | 17 [14, 20] | 0.072 |
| Maximum LV outflow tract gradient (mmHg) |  | 6 [4, 8] | 5 [4, 11] | 0.684 |
| LV end-diastolic diameter (mm) |  | 46 [40, 50] | 45 [41, 50] | 0.886 |
| LV ejection fraction (%) |  | 60 [55, 62] | 60 [55, 62] | 0.481 |
| LV diastolic dysfunction | Normal | 45 (63.4) | 24 (53.3) | 0.548 |
|  | Impaired relaxation | 15 (21.1) | 14 (31.1) |  |
|  | Pseudo-normalisation | 6 (8.5) | 5 (11.1) |  |
|  | Restrictive | 5 (7.0) | 2 (4.4) |  |
| LA diameter (mm) |  | 39 [34, 44] | 42 [38, 47] | 0.112 |
|  |  |  |  |  |
| **Outcomes at inclusion** |  |  |  |  |
| Phenotype | Phenotype-negative | 42 (33.3) | 17 (16.0) | **0.003** |
|  | HCM 13-14 mm | 8 (6.3) | 10 (9.4) |  |
|  | HCM >=15 mm | 76 (60.3) | 75 (70.8) |  |
|  | DCM | 0 (0.0) | 4 (3.8) |  |
| Primary outcome | Composite endpoint | 65 (50.8) | 50 (49.5) | 0.894 |
|  | Maximum wall thickness ≥20 mm | 51 (41.1) | 37 (38.1) | 0.680 |
|  | Septal reduction therapy | 12 (9.6) | 5 (5.3) | 0.311 |
|  | Malignant ventricular arrhythmia | 12 (9.4) | 9 (9.1) | 1.000 |
|  | Heart failure | 28 (23.0) | 19 (20.7) | 0.741 |
|  | Congestive heart failure | 14 (12.2) | 14 (15.4) | 0.543 |
|  | Systolic heart failure | 16 (13.9) | 17 (18.7) | 0.445 |

Clinical characteristics of included subjects at the time of inclusion, stratified by whether blood collection has been performed so far. Continuous data are shown as means ± standard deviation for normally distributed variables or median [interquartile range] for non-normally distributed variables. Dichotomous and categorical data are shown as counts (% of valid). P-values < 0.05 are depicted in bold. NYHA, New York Heart association; ESC HCM Risk-SCD calculator, European Society of Cardiology hypertrophic cardiomyopathy sudden cardiac death risk prediction calculator; LV, left ventricular; LA, left atrial; HCM, hypertrophic cardiomyopathy; DCM, dilated cardiomyopathy

**Figure S1: Lab protocol**

Protocol for the processing of samples for biobanking for later use in biomarker studies. Li, Lithium
